# Medical Society of the County of Chautauqua

**Published:** 1898-08

**Authors:** C. A. Ellis


### MEDICAL SOCIETY OF THE COUNTY OF CHAUTAUQUA.

Reported by C. A. ELLIS, M. D., Secretary.

THE annual meeting of the Medical Society of the County of Chautauqua was held at Chautauqua, N. Y., July 12, 1898, Dr. Morris N. Bemus, of Jamestown, presiding.

The business session was held at 11.30 a. m. The election of officers resulted as follows: President, Dr. M. N. Bemus, of Jamestown; vice-president, Dr. V. M. Griswold, Fredonia; secretary and treasurer, Dr. C. A. Ellis, Sherman; censors, Drs. J. Murphy, T. D. Strong, Wm. M. Bemus.

Dr. Wm. C. Krauss, president of the Central New York Medical Association, invited the society to become members of that association, which it accepted.

The society passed a resolution of sympathy for President Lewis Miller, of Chautauqua, whose son Theodore was killed at Santiago, the news having just been received.

The report of the board of censors showed that three persons who were practising illegally had been stopped by a notice from the chairman, Dr. Murphy, that he would prosecute them if they continued.

The report of the board of mid-wife examiners showed that the large majority applying for examination had been granted a certificate. It was also reported that this law had been a very profitable investment for the mid-wives, the certificate giving them a standing that resulted in a very large increase in the number of their cases.

The scientific session occupied the afternoon and evening and was well attended.

The subject of the president's annual address was A case of secondary hemorrhage caused by constipation. This paper was freely discussed and many valuable points were emphasised.

Dr. Wm. C. KRAUSS, of Buffalo, read a paper upon Hysteria and brain tumors. A discussion followed, during which a number of questions were propounded to Dr. Krauss.

A few cases were presented for diagnosis and treatment and a case of multiple fracture of jaw was shown with perfect results.

Dr. V. M. GRISWOLD, of Fredonia, gave a report of the recent small-pox out-break at that place and exhibited some photographs of the cases. There was no doubt as to the correctness of the diagnosis, it being a light form of small-pox.

In the absence of Dr. J. J. Mahoney the president called upon Dr. Thomas D. Strong, of Westfield, for some incidents he could recall of Admiral Dewey, the doctor at one time being his teacher. This was a happy surprise to most of the society. As usual the remarks of Dr. Strong were instructive and entertaining and he was heartily applauded.

The evening session opened at 8.00 p. m. Dr. C. C. Frederick, of Buffalo, read a paper upon The early diagnosis of malignancy in abdominal and pelvic disease. There is little doubt but that those who heard this paper will be still more careful in their future work, for the subject was very forcibly presented.

Dr. WM. SEAMAN BAINBRIDGE, of New York, read a paper upon Silver and silver salts in Surgery with special relation to wound treatment.

Dr. BAINBRIDGE has just returned from Europe and was the first American to enter and work with the German experimenters upon the subject which is promising so much in the antiseptic field.

The following were present: Dr. N. E. Beardsley, Dunkirk; Dr. M. N. Bemus, Jamestown; Dr. W. Seaman Bainbridge, of New York; Dr. J. J. Drake, Stockton: Dr. Wm. Casper Duke, Cassadaga; Dr. C. A. Ellis, Sherman; Dr. W. J. French, Hamlet; Dr. V. M. Griswold, Fredonia; Dr. J. J. Lenhart, Bemus Point; Dr. G. D. Marsh, Sherman; Dr. Macdonald Moore, Fredonia; Dr. J. W. Morris, Jamestown; Dr. Eliza M. Mosher, Ann Arbor, Michigan; Dr. James Murphy, Sherman; Dr. E. S. Rich, Kennedy; Dr. Nelson G. Richmond, Fredonia; Dr. E. A. Scofield, Bemus Point; Dr. O. C. Shaw, Kennedy; Dr. George F. Smith, Sinclairville; Dr. Thomas D. Strong, Westfield; Dr. B. S. Swetland, Brockton; Dr. W. D. Wellman, Jamestown; Dr. T. C. Wilson, Dewittville; Dr.

Wm. C. Krauss, Dr. C. C. Frederick, Buffalo; Dr. A. W. Dods, Fredonia; Dr. G. M. Lewis, Panama; Dr. J. R. Smith, Connewango Valley.

The meeting adjourned at 9.40 p. m., to meet at Fredonia, December 13, 1898.

